# The VELVET A Orthologue VEL1 of *Trichoderma reesei* Regulates Fungal Development and Is Essential for Cellulase Gene Expression

**DOI:** 10.1371/journal.pone.0112799

**Published:** 2014-11-11

**Authors:** Razieh Karimi Aghcheh, Zoltán Németh, Lea Atanasova, Erzsébet Fekete, Melinda Paholcsek, Erzsébet Sándor, Benigno Aquino, Irina S. Druzhinina, Levente Karaffa, Christian P. Kubicek

**Affiliations:** 1 Institute of Chemical Engineering, Research Division Biotechnology and Microbiology, Microbiology Group, Vienna University of Technology, 1060 Vienna, Austria; 2 Department of Biochemical Engineering, Faculty of Sciences and Technology, University of Debrecen, H-4032 Debrecen, Hungary; 3 Department of Human Genetics, Faculty of Medicine, University of Debrecen, H-4032 Debrecen, Hungary; 4 Faculty of Agricultural and Food Science and Environmental Management, Institute of Food Science, H-4032 Debrecen, Hungary; 5 Austrian Center of Industrial Biotechnology, c/o Institute of Chemical Engineering, Vienna University of Technology, 1060 Vienna, Austria; 6 Austrian Center of Industrial Biotechnology, 8010 Graz, Austria; University of Wisconsin - Madison, United States of America

## Abstract

*Trichoderma reesei* is the industrial producer of cellulases and hemicellulases for biorefinery processes. Their expression is obligatorily dependent on the function of the protein methyltransferase LAE1. The *Aspergillus nidulans* orthologue of LAE1 - LaeA - is part of the VELVET protein complex consisting of LaeA, VeA and VelB that regulates secondary metabolism and sexual as well as asexual reproduction. Here we have therefore investigated the function of VEL1, the *T. reesei* orthologue of *A. nidulans* VeA. Deletion of the *T. reesei vel1* locus causes a complete and light-independent loss of conidiation, and impairs formation of perithecia. Deletion of *vel1* also alters hyphal morphology towards hyperbranching and formation of thicker filaments, and with consequently reduced growth rates. Growth on lactose as a sole carbon source, however, is even more strongly reduced and growth on cellulose as a sole carbon source eliminated. Consistent with these findings, deletion of *vel1* completely impaired the expression of cellulases, xylanases and the cellulase regulator XYR1 on lactose as a cellulase inducing carbon source, but also in resting mycelia with sophorose as inducer. Our data show that in *T. reesei* VEL1 controls sexual and asexual development, and this effect is independent of light. VEL1 is also essential for cellulase gene expression, which is consistent with the assumption that their regulation by LAE1 occurs by the VELVET complex.

## Introduction

Cellulose and hemicelluloses form the major amount of plant biomass and thus represent the largest reservoir of renewable carbon sources on Earth, which could potentially replace fuels and refinery products derived from fossil carbon components [1]. To this end, efficient hydrolysis of the plant cell wall polymers to soluble oligo- and monomers is essential. The Sordariomycete *Trichoderma reesei* is most widely used for the industrial production of cellulolytic and hemicellulolytic enzymes and has become a basis for the modern paradigm of these enzymes [2]. The *T. reesei* genome encodes two cellobiohydrolases, five endo-ß-1,4-glucanases, and several ß-glucosidases, hemicellulases and accessory enzymes [3]. Most of these genes are regulated in a consistent manner, and are expressed only in the presence of an inducer, which can be either cellulose itself, disaccharides generated by its degradation (such as sophorose) or the galactosyl-β-1,4-glucoside lactose [3]–[7]. Today, seven transcription factors have been identified that participate positively or negatively in this regulation, of which XYR1 (xylanase regulator 1) is the main activator of both cellulase and hemicellulase gene expression [3], [8]. However, we have recently shown that the expression of genes for lignocellulose degradation in *T. reesei* is further obligatorily dependent on the function of the protein methyltransferase LAE1 [9], the orthologue of the *A. nidulans* regulator of secondary metabolism and development LaeA [10]. This regulation requires a functional *xyr1*, but a *lae1* loss-of-function cannot be rescued by *xyr1* overexpression [9], which would be consistent with the hypothesis that LaeA acts by removing the repressive chromatin [11]. However, a genome-wide analysis of H3K4 and H3K9 methylation patterns in *T. reesei lae1* mutants did not show any methylation changes at the cellulase loci [12]. More recently, the methyltransferase activity of *A. nidulans* LaeA was shown to exclusively perform automethylation of LaeA, but ironically just this automethylated methionine residue is not conserved in *T. reesei* [13]. Hence the mechanism of LAE1-dependence of cellulase gene expression remains enigmatic.

In *A. nidulans*, LaeA is known to be part of the trimeric VELVET protein complex, that consists of LaeA, VeA and VelB and that regulates secondary metabolism and development in *A. nidulans* [14]–[16], and pathogenesis on plants and humans in other genera [17]–[22]. Most studies on *veA* have been carried out in *Aspergillus* spp., where this gene has been described to control photodependent development, secondary metabolism and pathogenesis-associated processes [14]–[16]. Thorough genetic, molecular, and biochemical work has recently shown that Velvet is part of a high-molecular-weight complex containing at least 10 different proteins, some of which have been assigned distinct regulatory roles [15], [22]–[26]. The VeA orthologue VEL1 of *Trichoderma virens* has been shown to regulate sporulation, chlamydospore formation, secondary metabolite synthesis and mycoparasitism [27].

Our finding that LAE1 is essential for cellulase gene expression [9], independent of the underlying mechanism, raised the hypothesis that this function may require a functional VELVET complex. To test this hypothesis, we have therefore cloned and functionally characterized the ortholog of the central component of the VELVET complex, VeA, from *T. reesei* (VEL1; the gene/protein name was chosen in view of the Sordariomycete nomenclature, which uses three letters and a number instead of only letters to designate a gene). We will show that *T. reesei vel1* – like *lae1* – is essential for cellulase and hemicellulase gene expression. In addition, we will show that *vel1* is also essential for asexual and sexual development of *T. reesei* in a photo-independent manner.

## Results

### The VEL1 orthologue of *T. reesei*

The genome of *T. reesei* contains a single copy of the *vel1* gene (Trire2:122284; Gene Bank accession number of the respective protein VEL1: $EGR48103.1). The ORF of *vel1* consists of 1,801 bp, is interrupted by a single 79 bp intron and encodes a 574 amino acid protein. Inspection of the genome sequences of the improved cellulase producer strains QM 9414, NG14 and RUT C-30 [28], [29] showed that they contain gene copies with identical nucleotide sequences, proving that *vel1* has not been altered by mutagenesis towards improved cellulase formation.

A phylogenetic analysis of the *T. reesei* VEL1 protein sequence using the 50 best blastp hits from NCBI produced a tree whose shape was concordant with that of the species tree (data not shown), thus confirming earlier data [27]. Similarity of VEL1 to the VeA orthologues in *T. virens* and *T. atroviride* was consequently high (80 and 78% similarity over the entire amino acid sequence respectively). Highest identity outside of the genus was observed with *Nectria haematococca* (60%, 2e-180, 99% coverage), whereas it was only 36 and 38% with *A. nidulans* and *A. fumigatus*, respectively.

In accordance with studies in *A. nidulans* and *Neurospora crassa* [30]–[32], WoLF PSORT identified the protein to be able to enter the nucleus, the responsible motif being located at the N-terminus, and a leucine-rich nuclear import signal was putatively identified in the C-terminal quarter of the protein sequence. Like *A. nidulans* VeA, the *T. reesei* VEL1 protein also contains a potential PEST region (a sequence rich proline, serine, threonine and glutamine that indicates a short half-life; HAPPPLPPPPPSSYDAPPPAAR; PEST score 9.97), but in contrast to *A. nidulans*, where it is located at the C-terminal end of the protein [32], *T. reesei* VEL1 displays it in the middle of the protein immediately after the conserved N-terminal half (aa 290–311).

### *T. reesei vel1* transcript levels are carbon source dependent

**Figure 1:**
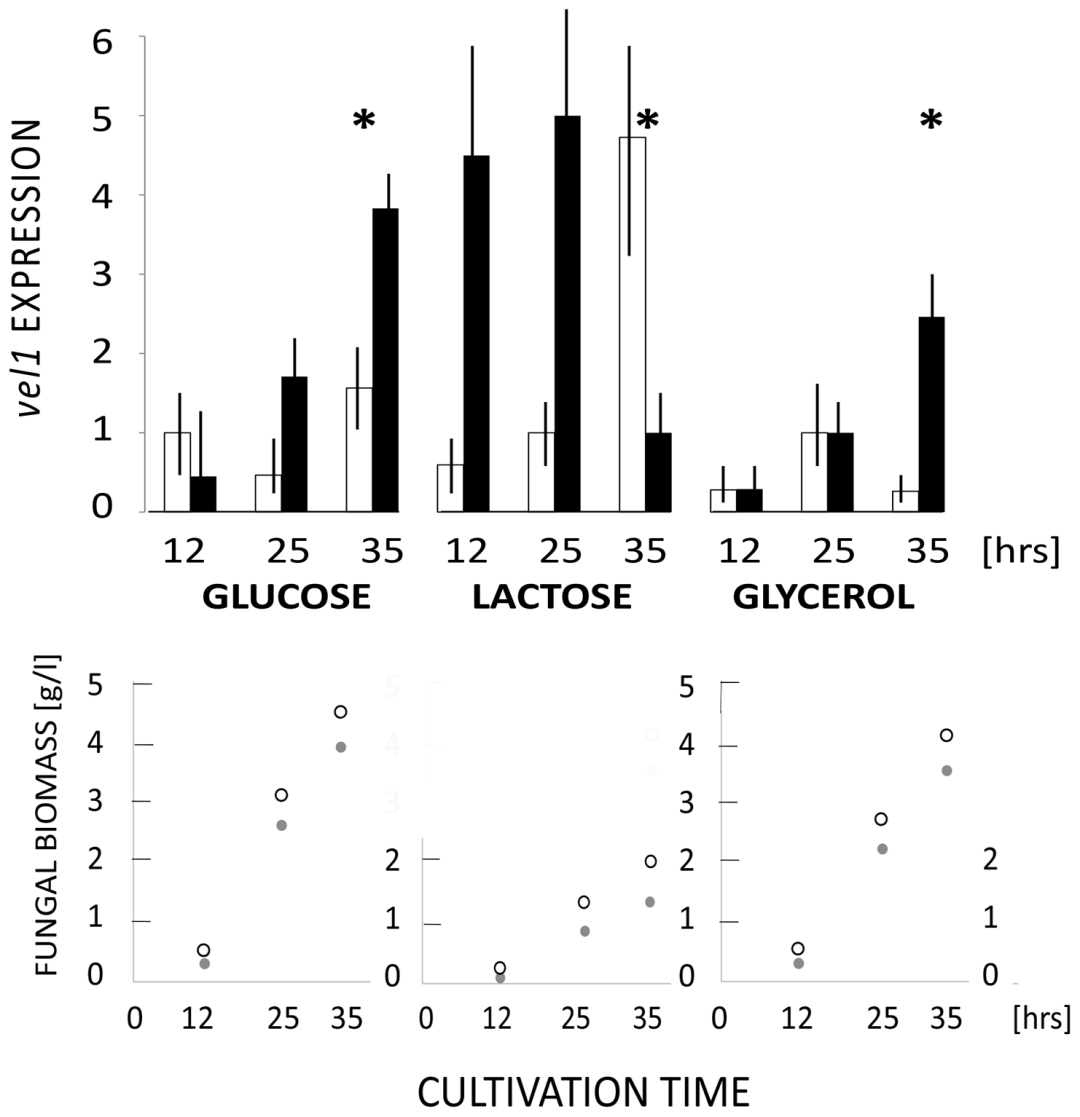
Effect of carbon source and light on *vel1* transcript levels in *T. reesei*. Transcript levels of *vel1* during growth on glucose, glycerol and lactose in *T. reesei* QM 9414 in the presence of ambient light (white bars) and darkness (full bars). Transcript levels are given in arbitrary units, which were calculated by normalizing the *vel1/tef1* ratio to that on glucose (12 h, ambient light). Data are means of at least 3 biological replicas. The asterisk indicates the time point where the cultures started to sporulate (this time point was the same in light and darkness).

We have examined *vel1* transcript levels during hyphal growth on plates and subsequent sporulation on three different carbon sources and in light and darkness. During growth on glucose and glycerol, two carbon sources that allow rapid growth of *T. reesei*, in the presence of light (empty bars), *vel1* mRNA was most abundant during the phase of most rapid growth (25 hrs) whereas it declined once growth ceased (Figure 1). During growth on lactose, which is much slower, the peak in *vel1* transcript accumulation in light occurred at 35 hrs, which again was the point where the growth rate was highest. During growth in darkness (full bars), the *vel1* transcript accumulated to much higher levels than under illumination (Figure 1). Again, the pattern on glucose and glycerol was similar, but now *vel1* mRNA levels increased with progressing time and were highest when growth already declined (35 hrs). During growth on lactose in the dark, however, *vel1* transcript levels were highest at the early time points but significantly decreased at 35 hrs. No correlation was observed between *vel1* mRNA levels and the onset of sporulation (asterisks in Figure 1). These data illustrate that the accumulation of *vel1*-mRNA expression is regulated by light and darkness, but that this regulation is further modulated by the carbon source in relation to the growth rate.

### VEL1 is required for normal hyphal tip growth, and essential for sexual and asexual development in *T. reesei*

To investigate the impact of *vel1* on the development of *T. reesei*, *vel1* null mutants (Δ*vel1*) were generated by replacing the *vel1* coding region with the *E. coli* hygromycin B phosphotransferase gene *hph* in the *T. reesei* QM 9414. In addition, we generated overexpressing (*vel1OE*) mutants by fusing the *vel1* ORF downstream of the strongly expressing *tef1* (elongation factor 1α- gene) promoter. We also attempted to retransform the wild-type *vel1* gene into the Δ*vel1* mutant, but even screening of more than 200 transformants did not yield a single stable one in which the wild type gene was integrated (data not shown). In order to identify true Δ*vel1* phenotypes, we therefore used three Δ*vel1* and two *vel1OE* strains for the investigations, and they yielded consistent results in all described cases. They had been purified and verified by PCR or Southern blotting analysis (Figure S1).

**Figure 2:**
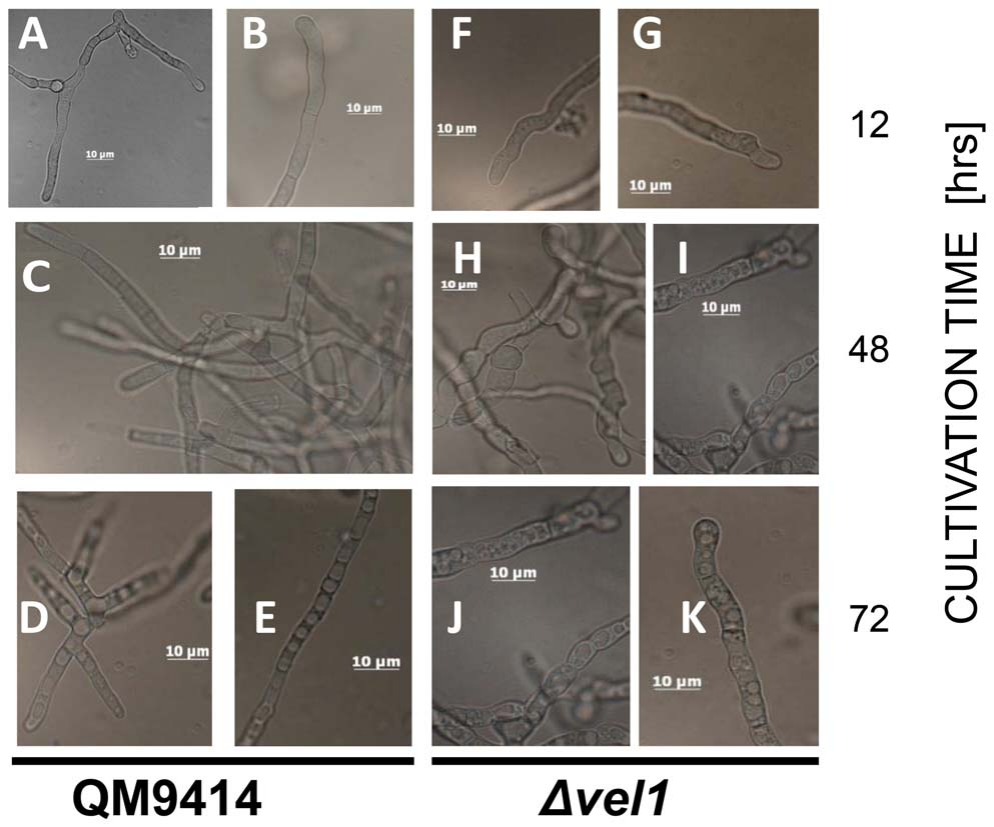
The impact of VEL1 on morphology of *T. reesei* in submerged culture. Morphology of growing hyphae of *T. reesei* QM9414 (A–E) and the Δ*vel1* (F–L) strains during submerged growth on Mandels-Andreotti medium with glycerol (1%, w/v) as a carbon source at 12 (A–B and F–G), 48 (C and H–I) and 72 (D–E and J–K) hours following inoculation.

The Δ*vel1* mutants displayed a distinct altered phenotype: when growing in submerged culture, the hyphae developed swollen, highly branched and much thicker hyphae (8–9 mm in diameter Figure 2 H and I) than the parent (3–4 mm; Figure 2 C). The hyphal tips of the Δ*vel1* mutants were often curved and filled with small vacuoles that increased upon aging of the cells (Figure 2 F,G and J, K).

**Figure 3:**
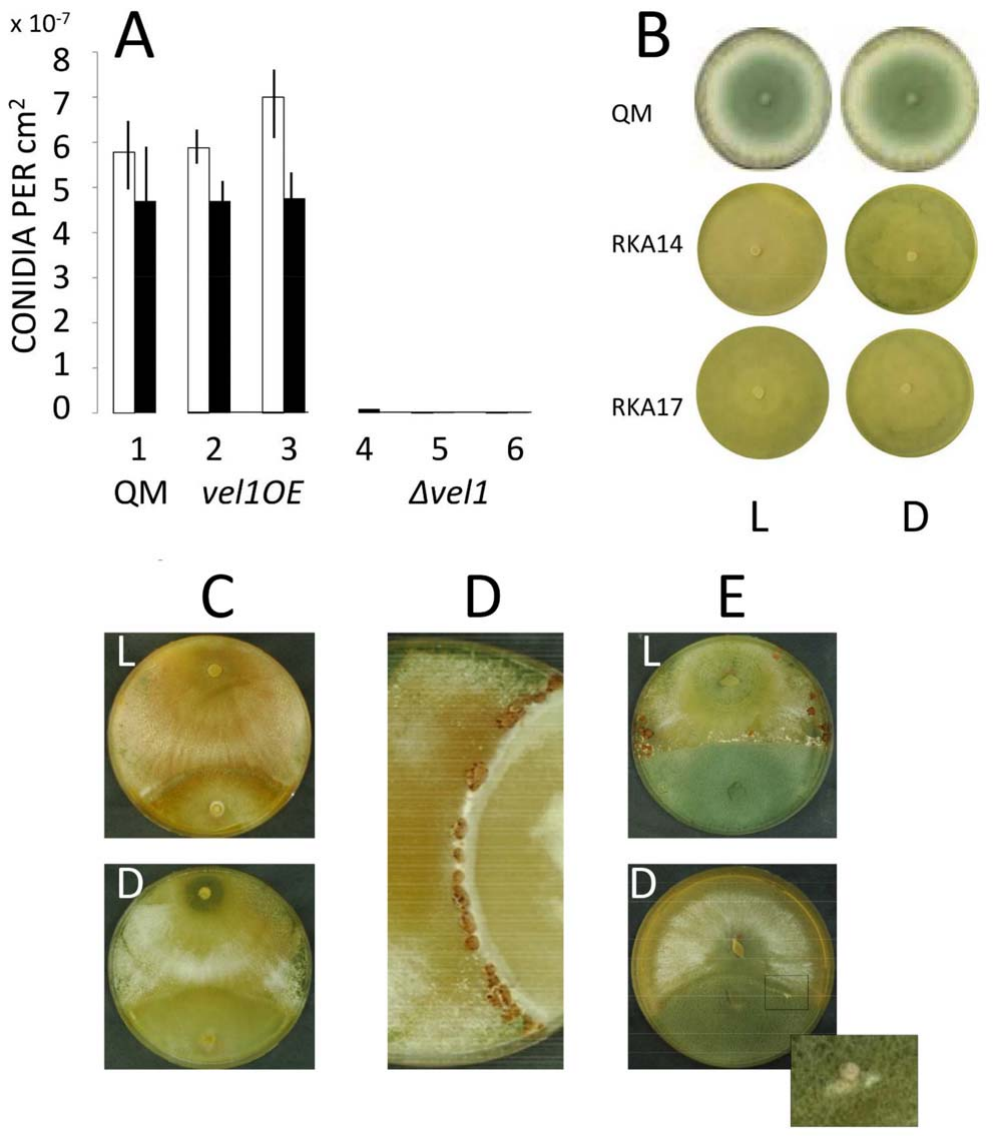
VEL1 is required for *T. reesei* development. (A) Effect of *vel1* on asexual development of *T. reesei*. Formation of conidia in the presence of ambient light (empty bars) and darkness (full bars) by *T. reesei* QM 9414 (1), in two *vel1OE* (2,3) and three *Δvel1* strains (4–6). Data are means of at least three independent biological experiments, and only results with p<0.05 are shown. (B) Phenotype of the parent strain and two *Δvel1* strains (RKA14 and RKA17; see Table 1) after 5 days of growth on PDA. (C) absence of fruiting body formation between *Δvel1* (top) and the compatible mating partner strain CBS 999.79 (bottom) in the presence of ambient light,L, and darkness, D. (D) control plate between QM 9414 (left) and CBS 999.79 (right) in the presence of ambient light. Note that fruiting in the wild-type does not occur in darkness [35], and thus no such plate is shown. (E) Fruiting body formation between *vel1OE* (bottom) and CBS 999.79 (top) under ambient light (L) or darkness (D). One of at least three replicate experiments is shown. The insert represents a magnification of the boxed part, highlighting the primordia.

On plates with glucose as a carbon source, the Δ*vel1* mutants exhibited a slightly lower growth and also the hyperbranched phenotype. More striking, however, was the completely impaired conidiation, both in light as well as in darkness (Figure 3A and B). Interestingly, the yellow pigment that is characteristic for *T. reesei* and not formed by Δ*lae1* strains [9], was still formed. Sporulation in the *vel1OE* strains appeared normal, as they formed conidia at a similar intensity as the parent strain in darkness, and only insignificantly stronger in the presence of light (Figure 3 A and B).

Sexual development of *T. reesei* (assayed by the formation of fertile perithecia) requires a functional VEL1 protein: the Δ*vel1* strain (*mat1-2*) did not produce any fruiting bodies when mated with the *T. reesei* tester strain CBS 999.79 (*mat1-1*; [33]) in the presence of ambient light, whereas the parent strain QM 9414 did (Figure 3 C). Since fruiting body formation in *T. reesei* obligatorily requires light [34], the lack of mating of *Δvel1* in darkness was the same as that of the parent strain. Interestingly however, the *vel1OE* strain was able to form primordia also in darkness, although with less frequency than in light (Figure 3 C). Yet these primordia lacked asci and were thus not fertile (data not shown).

### VEL1 is necessary for growth on cellulose and lactose

**Figure 4:**
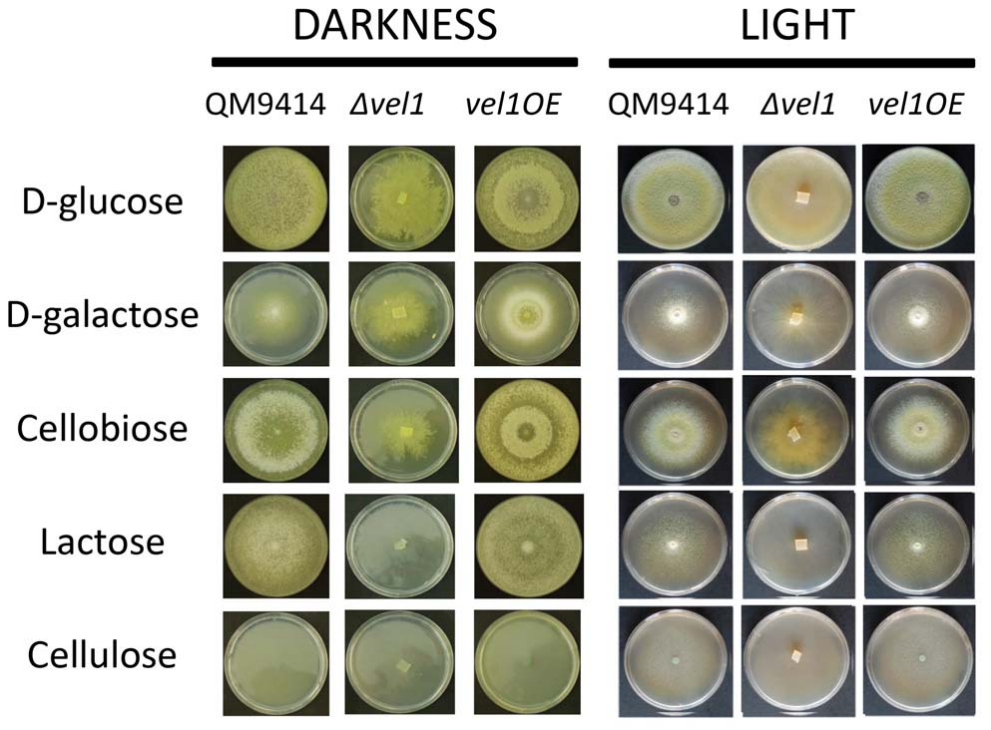
The impact of VEL1 on the utilization of different carbon sources by *T. reesei*. Effect of *vel1* on the growth of *T. reesei* on various carbon sources either in light or in darkness Photos were taken 5 days (120 h) after inoculation of the plates. Additional 2 days of cultivation did not alter the phenotypes shown.

The two *Δvel1* strains exhibited a slightly reduced growth on glucose as a carbon source on plates. To see whether this is a consequence of the alterations in hyphal morphology, or a different effect, we studied plate growth of *T. reesei* on several carbon sources including such related to cellulase formation. As can be seen (Figure 4), the slight reduction and altered hyphal phenotype was indeed observed on all carbon sources, and independent of growth in either light or in darkness. However, in addition to that, growth was significantly reduced on lactose and practically absent on cellulose. On the other hand, growth on lactose, cellobiose and cellulose was phenotypically normal in the *vel1OE* strain, and occurred at a faster rate. This suggests that, in addition to the effect on hyphal morphology, VEL1 obviously also impacts utilization of lactose and cellulose.

### VEL1 is essential for cellulase and hemicellulase transcript accumulation

**Figure 5:**
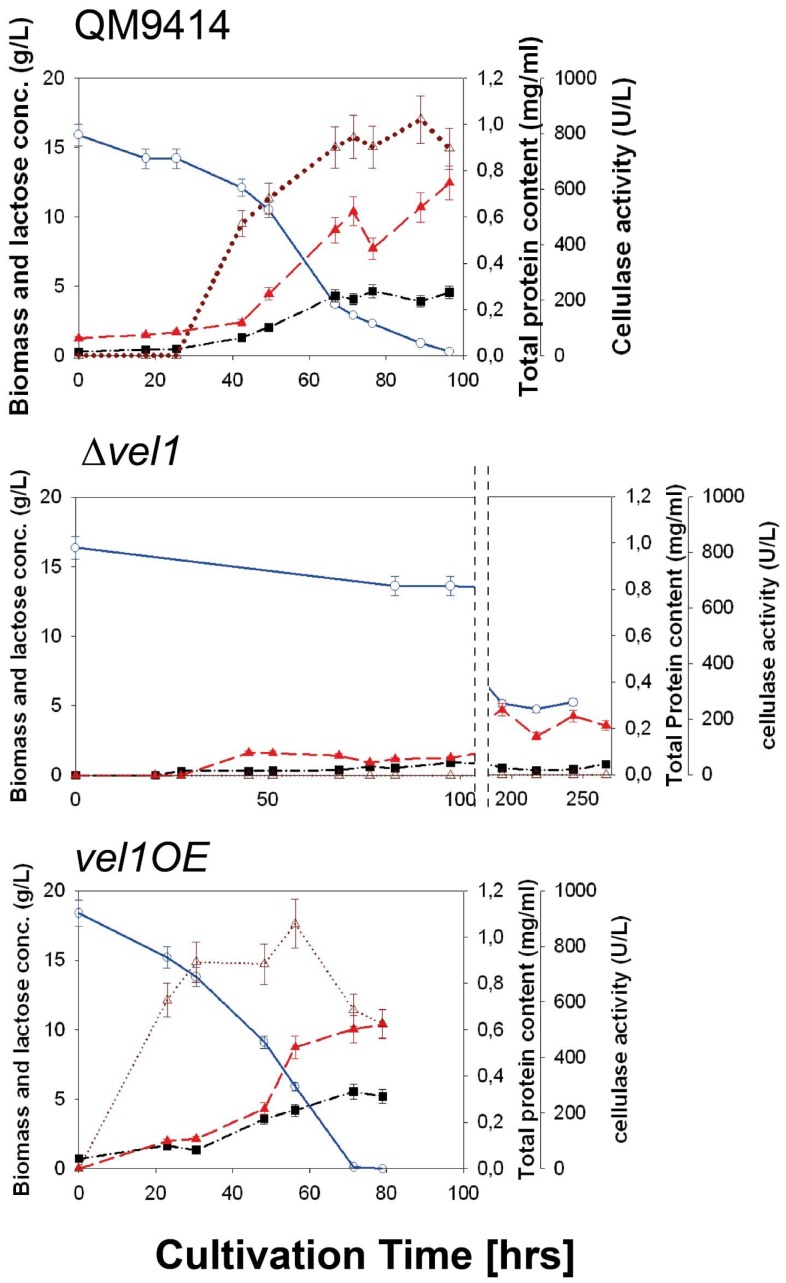
Impact of *vel1* deletion or overexpression on growth and cellulase formation by *T. reesei* on lactose. Cellulase formation by *T. reesei* QM 9414, a Δ*vel1* mutant and a *vel1OE* strain during growth on lactose. Only one strain is shown, but other strains with the same genotype gave consistent data. Data shown are means of at least two independent experiments and three measurements. Blue line and empty blue circles: lactose concentration; dark red dotted line and empty triangles: cellulase activity; bright red dashed line and full red triangles, extracellular protein concentration; black dashed line and black squares, fungal biomass concentration.

**Figure 6:**
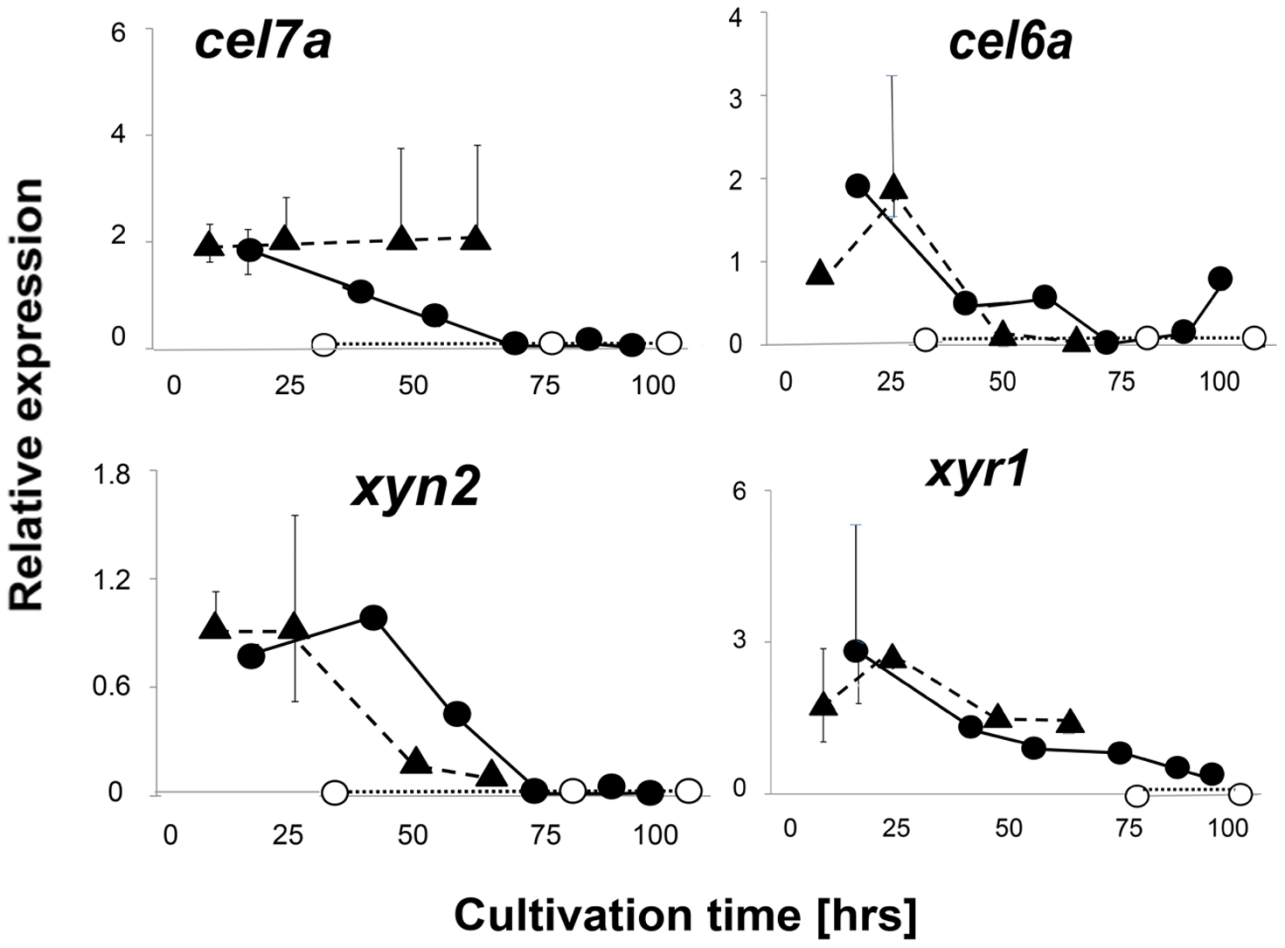
Impact of *vel1* deletion or overexpression on transcript levels of two cellulase, one hemicellulase and a cellulase regulator gene in *T. reesei* during growth on lactose. mRNA levels of two cellulase genes (*cel7a, cel6a*), one xylanase gene (*xyn2*) and the cellulase regulator *xyr1* in *T. reesei* QM 9414 (full circles and solid line), a *Δvel1* strain (empty circles and dotted line) and a *vel1OE* strain (full traingles and dashed line) under the fermentation conditions on lactose as shown in Figure 5. mRNA levels are given in arbitrary units, which were calculated by normalizing the ratio of the respective gene transcript to that of the housekeeping gene *tef1*, and relating all ratios to that obtained for the given gene at 17.5 hrs in *T. reesei* QM9414. Only one mutant strain is shown, but other strains with the same genotype gave consistent data. Data shown are means of at least two independent experiments and three measurements.

One hypothesis of this paper was that VEL1 would be necessary for the formation of cellulases in *T. reesei*. The above reported results with growth on cellulose were in accordance with this hypothesis. To test this directly, we first cultivated the parent and the two Δ*vel1* strains on lactose. The rationale for this was that lactose induces cellulase expression [6], but its utilization - in contrast to cellulose - is independent of the secreted cellulases [35]. As shown in Figure 5, submerged growth of the *Δvel1* mutants on lactose occurred - as on plates - at a much lower rate, and while the parent strain reached a final biomass concentration after 90–100 hrs of growth comparable mycelial dry weights of the two Δ*vel1* were only reached after 250 hrs of cultivation. Consistent with the lack of growth on cellulose of these mutants, no cellulase activity could be detected throughout the whole 250 hrs cultivation period (Figure 5 B). Also consistent with the faster growth of the *vel1OE* strain on lactose on plates, this strain also grew faster in submerged cultures on lactose, and started to produce cellulases much earlier (Figure 5 C). However, the final values of cellulase activity were only insignificantly higher in the *vel1OE* strain. Transcript data for the two major cellulase genes *cel7a* and *cel6a*, and the xylanase II-encoding gene *xyn2* perfectly supported the absence of cellulase activity in the two *Δvel1* mutants, indicating that the effect is on the level of gene transcription (Figure 6 A–C). Also the abundance of the transcript of *xyr1*, which encodes the major key cellulase and hemicellulase regulator XYR1, was strongly reduced in the two *Δvel1* mutants (Figure 6 D). This may also explain the impaired growth on lactose, because loss of function of *xyr1* also strongly impairs growth on lactose [35], [36]. In the case of the *vel1OE* strain, and also consistent with the above shown cellulose activities, the transcripts were not significantly higher than in strain QM 9414 but did not decrease that rapidly as in strain QM 9414 (Fig. 6 A–D).

In order to test the effect of *vel1* on cellulase formation under conditions where growth or inducer uptake are not affected, we used the ß-linked disaccharide sophorose and resting mycelia pregrown under non-inducing conditions (glycerol). Sophorose is a very powerful inducer of cellulases and xylanases in *T. reesei* under these conditions [3], [37] and the *ctr1* gene that encodes the sensor mediating sophorose induction is still expressed at a high level in a *T. reesei* mutant with impaired *Δxyr1* function [37]. This experiment should thus enable to detect effects of *vel1* without disturbance by effects of *vel1* on growth and nutrient uptake.

**Figure 7:**
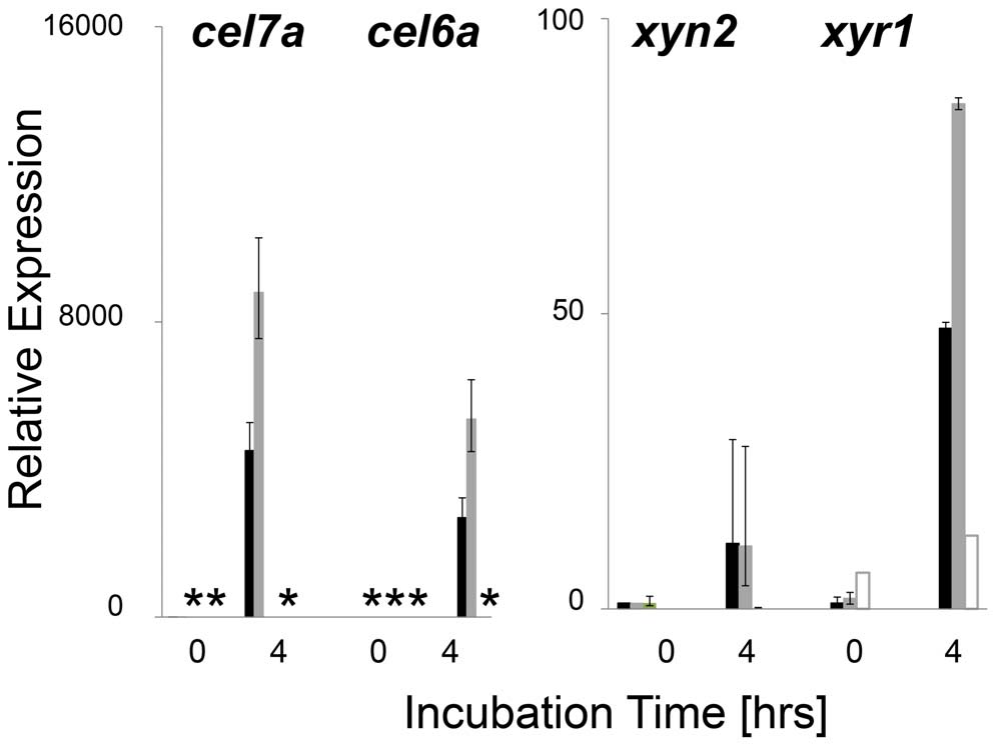
Impact of *vel1* deletion or overexpression on transcript levels of two cellulase, one hemicellulase and a cellulase regulator gene in *T. reesei* upon induction by sophorose. Transcript levels of two cellulase genes (*cel7a, cel6a*), one xylanase gene (*xyn2*) and the cellulase regulator *xyr1* in *T. reesei* QM 9414 (full symbols), a *vel1* strain (empty symbols) and a *vel1OE* strain (grey symbols) upon induction of mycelia, pregrown on glycerol for 20 hrs, by sophorose for 4 hrs. Transcript levels are given in arbitrary units, as specified in legend to figure 6. The asterisks indicate cases were the relative mRNA level was below the limit of detection. Only one mutant strain is shown, but other strains with the same genotype gave consistent data. Data shown are means of at least two independent experiments and three measurements.

As can be seen in Figure 7, addition of sophorose led to a strong increase in the relative abundance of the *cel7a, cel6a, xyn2* and *xyr1* transcripts with 4 hrs in the parent strain, and – with the exception of *xyn2* - roughly doubled in the *vel1OE* mutant. whereas they remained barely detectable in the *Δvel1* mutant. Also the accumulation of the *xyr1* transcript was strongly reduced, yet clearly detectable and notably present in higher amounts than in the parent strain at t = 0. This correlates with its partially constitutive expression. We conclude therefore that a loss of *vel1* function impairs cellulase gene expression, and VEL1 is thus essential for this process. It also shows that upregulation of *vel1* transcript levels increases cellulose gene transcription.

## Discussion

In this paper, we have explored the function of the velvet gene *vel1* of *T. reesei*. While this global regulator is conserved in Pezizomycota, studies of *veA* orthologs across several fungal genera have now established a significant diversity in its impact on fungal development [14], [16], [17]. The most striking example is asexual sporulation, a trait influenced by VeA/VelA/VEL1 in all fungi: whereas a *veA/velA* knock out in *A. nidulans, P. chrysogenum* and *N. crassa* increases conidiation [29], [38], [39], it results in decreased conidiation in the corresponding knock-out mutants of *A. fumigatus*, *A. parasiticus*, *A. flavus, Fusarium fujikuroi*, *F. graminearum, Dothistroma septosporum* and *T. virens* [19], [22], [27], [39]–[44]. Our study shows that *T. reesei* belongs to the second group as the *vel1* knock-out strains almost completely lacked conidiation. It is further interesting that this impairment was independent on the presence or absence of light, a finding so far only recently found in *A. fumigatus* [41]. Yet this coincides with the findings that the *T. reesei* parent strain forms the same number of conidia upon illumination as in the dark, and suggests that, differently from *A. nidulans* [38], light is not a relevant factor in the *veA/vel1*-mediated regulation of conidition in *A. fumigatus* and *T. reesei*.

Another example where the function of VEL1 differs from that of its ortholog in other fungi, is the complete loss of sexual development of *T. reesei Δvel1* mutants. Opposite findings have been reported for *N. crassa* [30], and while a similar elimination of sexual reproduction has been reported for *A. nidulans* [31], the effect of light on the action of *veA/vel1* is reversed: fruiting body formation in *T. reesei* only occurs in light [34] but in *A. nidulans* only in the darkness [37]. Interestingly, overexpression of *vel1* under a constitutive, light-independent promoter (*tef1*) enabled *T. reesei* to form some sterile primordia in darkness, an ability not shown by the wild type strains [34]. Thus an increased expression of *vel1* in the dark cannot overcome the inability of *T. reesei* to initiate full fruiting body formation, but can only initiate an early stage, suggesting that *vel1* controls several steps in this process in different ways. Thus, while *vel1* is essential for sexual development in *T. reesei*, it is only partially - if at all - responsible for its dependence on light. Consequently, while we have shown that VEL1 also regulates developmental processes in *T. reesei*, its mode of action and its interaction with environmental triggers remains unclear and cannot be deduced from analogy with other fungal systems.

On the other hand, there are also effects that appear to be conserved in all VeA/VelA/VEL1 orthologs, such as the consequences of their knock out on hyphal morphology, which are reflected in higher branching and shorter filaments caused by changes in cell wall metabolism [45]. Our findings of crippled, highly branched and thickened hyphae correlate well with respective findings in *P. chrysogenum, A. chrysogenum* and *Fusarium* spp. [40], [41], [44], [45].

While the impact of the velvet complex on the regulation of secondary metabolite production has been well documented in numerous fungi (for review see [14], [16]), only two papers have so far reported the participation of *veA/velA* in the regulation of extracellular enzyme synthesis: Kamerewerd et al. [46] reported that the class V chitinase PcchiB1 of *P. chrysogenum*, which is involved in cell wall turn-over, is strongly downregulated in a delta-*velA* mutant. And while this paper was in preparation, Duran et al. [47] reported that the expression of amylase and protease activity in *A. flavus* is impaired in a *veA* mutant, while an alpha-amylase was produced in greater quantities.

We have recently shown that LAE1 strongly impacts cellulase gene transcription in *T. reesei* and cellulase expression is completely abolished in *lae1* loss-of-function mutants [9]. Since LAE1 and VEL1, as in other fungi, can physically interact in *T. reesei* [12], we assumed that cellulase expression would also be affected by *vel1*. In this paper, we now provide evidence that the expression of cellulases in *T. reesei* is also completely dependent on a functional *vel1* gene, which agrees well with the stringent requirement for a functional *lae1* gene [9], and suggests that cellulase expression is indeed regulated by the velvet complex. The extension of our findings to all cellulases and hemicellulases appears justified because they are corregulated by sophorose and lactose [4]–[8], and thus - although we quantified only the transcripts of two cellulases and one xylanase - it can be safely assumed that the others are affected in the same manner too. In addition, we have shown that also the transcript levels of the central regulator of cellulase and hemicellulase biosynthesis, XYR1, is strongly reduced albeit not eliminated. This fits perfectly to the results with the *T. reesei Δlae1* mutant, in which also a low level of *xyr1* transcript levels was still observed, and consequently the expression of *xyr1* under a constitutive promoter did not rescue the *Δlae1* phenotype [9].

Taken together, the above and our study show that the regulatory targets of the velvet complex reach beyond mere secondary metabolism and development. The extension of the function of the velvet complex towards formation of extracellular hydrolytic enzymes is intriguing, because a regulatory interaction of secondary metabolism and fungal development is already well established [48], whereas such a link with hydrolases is so far unknown. However, in *T. reesei* a link between asexual sporulation and cellulase formation has recently been demonstrated [49]. This presence of cellulases and other plant cell wall hydrolases on the spores of the fungus was interpreted in terms of an advantage because the disperged spores could instantly initiate growth when arriving at a new substrate. In this regards it is also interesting that many of the cellulase genes of *T. reesei* are clustered in the genome with genes encoding secondary metabolite synthases [1], and indeed several of them were demonstrated to be expressed under conditions inducing cellulase biosynthesis [6], [7], [50]. However, the regulator coordinating sporulation, secondary metabolite formation and cellulase gene expression in *T. reesei* has not yet been identified. It is not XYR1, because *Δxyr1* mutants do not form cellulases but are still able to sporulate [49]. Likewise aconidial mutants of *T. reesei* still form cellulases (R. Linke and C.P. Kubicek, unpublished data), and thus the two processes are linked but not dependent on each other. This also agrees with the findings that the strongly reduced conidiation in the *Δlae1* strain of *T. reesei* is *xyr1* independent [9].

Based on the data of this paper, we therefore propose that the velvet complex is a superimposed regulatory level that coordinates the expression of cellulases, and secondary metabolites with asexual development in *T. reesei*. We also conclude from the above data that this coordination is not influenced by light, and this claim is further supported by the findings that cellulase and secondary metabolite gene expression is not significantly different (<1.5-fold at p<0.05; [51]) in *T. reesei* wild-type strains growing on cellulose under either illumination or in darkness. The identification of the signal triggering this coordinated expression and how it interacts with the velvet complex will be a challenge for further work, and may also identify new regulatory levels for potential further improvement of cellulase producing strains of *T. reesei*.

## Materials and Methods

### Strains and cultivation conditions

*T. reesei* strains used throughout this work are listed in Table 1. They were maintained on potato dextrose agar (PDA). *Escherichia coli* JM109 (Promega, Madison, Wisconsin) was used for plasmid construction and amplification. Cultures were grown at 28°C in a Sanyo incubator containing a Philips-master light source (TLD-15 W/840), either with continuous illumination (light conditions) or double wrapped in foil (dark conditions).

**Table 1:** Strains used in the present work.

Strain name	Genotype	Reference
QM 9414	*mat1-2*	[33]
CBS 999.79	*mat1-1*	[33]
RKA14	*Δvel1/mat1-2*	this work
RKA12	*vel1OE, mat1-2*	this work
RKA13	*vel1OE, mat1-2*	this work
RKA17	*Δvel1/mat1-2*	this work
RKA18	*Δvel1/mat1-2*	this work

Cultivations on lactose were carried out in 20 L stainless steel bioreactors (Zolend Ltd., Debrecen, Hungary) as described previously [52], using Mandels-Andreotti medium [53], except that agitation was 400 rpm. Induction experiments with sophorose were performed as described [54], using 20 h old mycelia pregrown on Mandels-Andreotti medium with glycerol (1%, w/v) as a carbon source.

Growth tests on plates were performed on Mandels-Andreotti medium, solidified with 2% (w/v) agar, but without peptone, and the carbon source indicated (1%, w/v).

### Nucleic acid isolation and hybridization

Fungal mycelia were harvested by filtration, washed with distilled cold water, frozen and ground under liquid nitrogen. For extraction of genomic DNA, plasmid DNA and RNA, purification kits (Wizard Genomic DNA Purification Kit, PureYield Plasmid Midiprep System and RNeasy plant kit, respectively, all from Promega) were used according to the manufacturer's protocol. Standard methods were used for electrophoresis, blotting and hybridization of nucleic acids.

### Construction of *T. reesei* recombinant strains

To study the function of VEL1, we constructed *T. reesei* strains in which *vel1* was deleted and strains, which *vel1* was expressed under the strong constitutive expression signals of the *tef1* (translation elongation factor 1-alpha encoding) promoter region [55].

To delete the *vel1* gene of *T. reesei*, the 1.8-kb *vel1* coding region was replaced by the *E. coli* hygromycin B phosphotransferase (*hph*) gene. This was performed by amplifying around 1.2-kb of the up- and downstream non-coding region of *vel1* from genomic DNA of *T. reesei* QM 9414 using the primer pairs given in Table S1. The two resulting PCR fragments were digested with *ApaI*/*Xho*I (upstream region) and *XhoI*/*Cla*I (downstream region) and ligated into a *Apa*I/*Cla*I restricted vector pBluescript SK(+) (Stratagene, La Jolla, California), followed by the insertion of the 2.4-kb *Sal*I*/XhoI* fragment of the *hph* gene into the *Xho*I site resulting in pRKA_D122284hph.

For expression of *vel1* under a strong constitutive promoter, a 2,275-bp *vel1* PCR fragment including the coding and terminator region was amplified with the oligonucleotides Fw_ Ptef1:vel1_Cla1 and tef1:vel1-HindIII (Table S1) and then inserted downstream of the *tef1* promoter region [56] into the ClaI/HindIII sites of pLH1hphtef1 resulting in vector pRKA-OE122284hph, which contains the *E. coli* hygromycin B phosphotransferase (*hph*) under *T. reesei* expression signals as selection marker [57].

All vectors constructed were verified by nucleotide sequencing.

### Fungal transformation

Protoplast preparation and DNA mediated transformation was performed as described [58]. The strains were purified twice for mitotic stability, and integration of the expression cassettes was verified by PCR analysis. Gene copy numbers of the integrated constructs were determined by Southern analysis [59], using chromosomal DNA cleaved with *BamHI/HindIII*.

### Analysis of sexual and asexual development

For sexual reproduction, *T. reesei* parent and mutant strains and the compatible mating partner strain CBS 999.79 [34] were pre-grown on PDA for 4 days, and agar culture plugs then transferred on fresh PDA (Difco, Lawrence, KS, USA) on opposite sides of the plate at a 1 cm distance from the edge. The plates were kept at room-temperature and exposed to day light or kept in complete darkness for 4–7 days (see above). All pairs of strains which formed fruiting bodies were visually inspected until the maturation stage was achieved and ascospores were dispersed. Monoascospore cultures were isolated by dispersing the solution with a cotton swab on multiple PDA plates. After overnight incubation several single germinated spores were selected with an aid of a stereomicroscope, transferred to a new PDA plate and cultivated at 28°C.

To test for photodependent conidiation, each PDA plate was inoculated with a 5-mm diameter mycelial plug taken from the edge of a 3-day-old colony. Three replications were done for each treatment. Plates were incubated at 28°C for 8 days in either complete darkness and cycles of 12 h illumination/12 h darkness (see above), and conidia then harvested by gently rubbing them off in an equal volume of physiologically salt (1%, w/v, Tween and 0.8% w/v NaCl), filtering through glass wool, and centrifugation (5000× g, 10 min). The conidia were then suspended in 2.5 g/l phytagel (Phytagel, SIGMA, Steinheim, Germany), mixed and their transmission measured at 590 nm in a Biolog standard turbidimeter. The number of conidia was calculated using a calibration curve with *T. reesei* conidia.

### Enzymatic assays and determination of fungal dry weight

Cellulase enzyme activities were determined using carboxymethylcellulose (1%, w/v) [56]. Protein in the culture supernatant was determined by the method of Bradford [60]. Fungal dry weight was determined by filtering an aliquot of the culture through glass sinter funnels (porosity G1), washing with tap water and drying at 80°C to constant weight. The lactose concentration in the fermentor was determined by HPLC as described earlier [52].

### Gene expression by quantitative PCR

DNase treated (DNase I, RNase free; Fermentas) RNA (5 µg) was reverse transcribed with the RevertAid First Strand cDNA Kit (Fermentas) according to the manufacturer's protocol with a combination of oligo-dT and random hexamer primers. All qPCR assays were performed on a Bio-Rad (Hercules, CA) iCycler IQ. For the reaction the IQ SYBR Green Supermix (Bio-Rad, Hercules, CA) was prepared for 25 µl assays with standard MgCl_2_ concentration (3 mM) and a final primer concentration of 100 nM each. All assays were carried out in 96-well plates. The amplification protocol consisted of an initial denaturation step (3 min at 95°C) followed by 40 cycles of denaturation (15 sec at 95°C), annealing (20 sec; for primers and the respective temperature see Table S2) and elongation (10 sec at 72°C). Determination of the PCR efficiency was performed using triplicate reactions from a dilution series of cDNA (1; 0.1; 0.01; 0.001). Amplification efficiency was then calculated from the given slopes in the IQ5 Optical system Software v2.0. Expression ratios were calculated using REST Software [61]. All samples were analyzed in at least two independent experiments with three replicates in each run.

### Bioinformatic analysis

Identification of PEST regions (protein domains that are enriched in proline, glutamic acid, serine, and threonine residues) that may lead to rapid protein degradation, typical for unstable proteins, was performed with epestfind ([62]; http://emboss.bioinforatics.nl/cgi-bin/emboss/epestfind). The cellular localization of proteins was analyzed by WoLF PSORT (Protein Subcellular Localization Prediction tool; [63], http://wolfpsort.org/), and leucine-rich nuclear export signals (NES) identified by NetNES 1.1 Server ([64]; http://www.cbs.dtu.dk/services/NetNES/).

## Supporting Information

Figure S1 **Verification of the recombinant** ***T. reesei*** **strains.** PCR verification of *vel1* knock out in *T. reesei*: (A) structure of the disrupted (top) and native *vel1* locus (below). Numbers indicate the size (in kb) of the respective areas. The dotted line defines the gene construct present in the deletion cassette. The arrows ***a–d*** specify the primers used for amplification the homologous integrated knock-out construct (a and b; result shown in B), and of the native *vel1* gene (c and d; result shown in C), respectively. ***a***, pVel1; ***b***, hph_int; ***c***, Vel_int1; ***d***, Vel_int2 (for sequences see Table S2). Tracks: 1, parent strain QM9414; 2, *Δvel1* strain RKA14, 3, *Δvel1* strain RKA17, *Δvel1* strain RKA18. Southern analysis: D, scheme of the wild-type *vel1* locus. DNA was cleaved by HindIII and BamHI, and hybridization was done by a full-length 1.8 probe of *vel1*. E, resulting autoradiograph: Tracks: 1, RKA12; 2, RKA13; 3, parent strain QM9414; 4, size marker ladder.
(DOCX)

Table S1 **Oligonucleotide primers used in this work.**
(XLSX)

Table S2 **qPCR primers used in this work.**
(XLSX)
