# Supplementary material for: The VELVET A Orthologue VEL1 of Trichoderma reesei Regulates Fungal Development and Is Essential for Cellulase Gene Expression
Source: PLoS One. 2014 Nov 11;9(11):e112799. doi: 10.1371/journal.pone.0112799 (PMC4227869; doi:10.1371/journal.pone.0112799)
Supplement: Figure S1 — Verification of the recombinant T. reesei strains. PCR verification of vel1 knock out in T. reesei: (A) structure of the disrupted (top) and native vel1 locus (below). Numbers indicate the size (in kb) of the respective areas. The dotted line defines the gene construct present in the deletion cassette. The arrows a–d specify the primers used for amplification the homologous integrated knock-out construct (a and b; result shown in B), and of the native vel1 gene (c and d; result shown in C), respectively. a, pVel1; b, hph_int; c, Vel_int1; d, Vel_int2 (for sequences see Table S2). Tracks: 1, parent strain QM9414; 2, Δvel1 strain RKA14, 3, Δvel1 strain RKA17, Δvel1 strain RKA18. Southern analysis: D, scheme of the wild-type vel1 locus. DNA was cleaved by HindIII and BamHI, and hybridization was done by a full-length 1.8 probe of vel1. E, resulting autoradiograph: Tracks: 1, RKA12; 2, RKA13; 3, parent strain QM9414; 4, size marker ladder. (DOCX) [file pone.0112799.s001.docx]

**Supplementary Figure S1:**

**Verification of the recombinant *T. reesei* strains**

PCR verification of *vel1* knock out in *T. reesei*: (A) structure of the disrupted (top) and native *vel1* locus (below). Numbers indicate the size (in kb) of the respective areas. The dotted line defines the gene construct present in the deletion cassette. The arrows ***a – d*** specify the primers used for amplification the homologous integrated knock-out construct (a and b; result shown in B), and of the native *vel1* gene (c and d; result shown in C), respectively. ***a***, pVel1; ***b***, hph_int; ***c***, Vel_int1; ***d***, Vel_int2 (for sequences see Table 2). Tracks: 1, parent strain QM9414; 2, *∆vel1* strain RKA14, 3, *∆vel1* strain RKA17, *∆vel1* strain RKA18. Southern analysis: D, scheme of the wild-type *vel1* locus. DNA was cleaved by HindIII and BamHI, and hybridization was done by a full-length 1.8 probe of *vel1*. E, resulting autoradiograph: Tracks: 1, RKA12; 2, RKA13; 3, parent strain QM9414; 4, size marker ladder.
